# Dextroamphetamine-Amphetamine Augmentation in the Treatment of Treatment-Resistant Depression

**DOI:** 10.7759/cureus.27755

**Published:** 2022-08-07

**Authors:** Tyler M Small, Sanket Dhat, Zeeshan Faruqui

**Affiliations:** 1 Psychiatry, Liberty University College of Osteopathic Medicine, Lynchburg, USA; 2 Psychiatry, Liberty University, Lynchburg, USA; 3 Psychiatry, Centra Medical Group, Lynchburg, USA; 4 Department of Psychiatry, Keystone Behavioral Health, Chambersburg, USA

**Keywords:** suicidal ideations, treatment-resistant depression, tms, adderall, major depressive disorder (mdd)

## Abstract

There is much debate over a precise definition of treatment-resistant depression (TRD) as well as the method of staging this illness. Although there is some non-consensus on a definition for TRD, the most widely accepted definition of TRD is a failure to achieve clinical improvement of depressive symptoms following a trial of two or more antidepressant medications from two or more different pharmacological classes at adequate dosage, duration, and compliance. Some sources lower the threshold to failure of one medication, but most support two medications. Although both men and women can be effected by TRD, our review found a slight predominance in older women. Here we present a 62-year-old female diagnosed with severe major depressive disorder that meets the criteria for treatment-resistant depression. This patient failed to experience consistent relief of symptoms using different antidepressant monotherapies as well as different combinations of therapies. Transcranial magnetic stimulation provided a brief relief of symptoms in this patient; however, relapse occurred a few months later. This case is unique as this patient has recently experienced significant relief of her depressive symptoms using amphetamine and dextroamphetamine (Adderall) as an adjunct to her antidepressant therapy. We will review the literature that currently exists on treatment-resistant depression and the treatment options for TRD, as well as present our case. To our knowledge, a case of TRD responding so strongly to Adderall after failing to respond to such drastic pharmacologic measures, as well as TMS, has not been reported.

## Introduction

According to the National Institute of Mental Health, an estimated 17.3 million Americans over the age of 18 experienced at least one major depressive episode in 2017 [1]. It has been reported that only 29-46% of patients diagnosed with depression and treated with antidepressant monotherapy achieve clinical improvement of their symptoms [2-4], creating a clinical problem when trying to treat patients suffering from depression. The term “treatment-resistant depression (TRD)” was first used in 1974 following the World Health Organization conference on major depressive disorder (MDD) [5]. Currently, the most widely accepted definition of TRD is a failure to achieve clinical improvement of depressive symptoms following a trial of at least two antidepressant medications from two different pharmacological classes at adequate dosage (which may vary from patient to patient), duration (at least four to eight weeks), and compliance. It is estimated that 10-29% of patients diagnosed with MDD also meet the criteria for TRD. It has been found that TRD is slightly more prevalent among women and effects patients between 36 and 64 years of age [6]. The treatment options for TRD are numerous and include both pharmacologic and non-pharmacologic therapies. The treatment options discussed in this literature review are not an exhaustive list.

Pharmacological therapy

Some of the most used antidepressants include selective serotonin reuptake inhibitors (SSRIs), serotonin, norepinephrine reuptake inhibitors (SNRIs), monoamine oxidase inhibitors (MAOIs), and tricyclic antidepressants (TCAs). These drugs can be used as monotherapy or in combination for more severe cases. The combination of a TCA and an SSRI has been found to be more effective than either drug class alone, and the addition of moclobemide (MAOI) to the TCA and SSRI combination is even more effective. It must be noted, however, that this three-drug combination is not well tolerated by most patients, and a TCA/MAOI combination is generally not recommended. The use of SSRIs like fluoxetine with either desipramine (TCA), trazodone, or mirtazapine has also been shown to be efficacious [7].

The literature we reviewed shows that augmentation methods using non-antidepressant medications in the treatment of TRD have been well documented. Lithium, a mood stabilizer, has been shown to be an effective augmentation medication to classic antidepressant therapy. The side effect profile of lithium must be strongly considered, especially before prescribing to a patient with other augmentation methods still available. Pindolol, a non-selective beta blocker typically used in the management of hypertension, is also theorized to agonize serotonin receptors. The literature surrounding the use of pindolol in the treatment of TRD is both positive and neutral when compared to placebo. Typical and atypical antipsychotics have been used as augmentation therapy in the treatment of TRD, and it has been found that patients who received an SSRI or SNRI along with an atypical antipsychotic, specifically aripiprazole and quetiapine, were 1.69 times more likely to experience a clinical response to treatment than those who received placebo. The use of anticonvulsants, most commonly lamotrigine, in the management of TRD is very well supported by the literature we reviewed [7].

The relationship between thyroid dysfunction and depressive symptoms has long been established, and our literature review found that euthyroid patients receiving augmentation with T3 were two times more likely to experience relief of depressive symptoms than patients who did not. The use of ketamine, an N-methyl D-aspartate (NMDA) receptor antagonist, in the management of TRD is currently supported by one systematic review, multiple case reports, and multiple randomized clinical trials, all showing safe and efficacious use of ketamine as an augmentation to antidepressant regimens. Other agents such as bupropion, amantadine, and pramipexole have all proven to be efficacious in the treatment of TRD when used as the augmentation to traditional antidepressant therapy.

The literature regarding the augmentation of antidepressant therapy with psychostimulants in the treatment of TRD mainly revolves around the use of methylphenidate (Ritalin) [8,9], dextroamphetamine, dextroamphetamine-amphetamine (Adderall), lisdexamphetamine (Vyvanse), atomoxetine, and modafinil. The use of stimulants such as methylphenidate and dextroamphetamine has been well documented and our literature search found one uncontrolled study, two case reports, a randomized, double-blind clinical trial, and a series of studies [10] involving elderly patients, all showing favorable outcomes. The literature surrounding the use of atomoxetine in the management of TRD [11] is both neutral and positive. We also found that patients who received modafinil, armodafinil, or lisdexamphetamine in addition to their established antidepressant regimen [12] were more likely to report remission of depressive symptoms than patients receiving augmentation with placebo.

Nonpharmacological therapy

The nonpharmacologic therapies that are currently documented in the treatment of TRD include electroconvulsive therapy (ECT), transcranial magnetic stimulation (TMS), deep brain stimulation (DBS), vagus nerve stimulation (VNS), and psychotherapy. ECT has been proven to be capable of relieving TRD symptoms [9], but those effects have not been shown to last longer than 55 weeks in most patients [13]. TMS has been shown to produce relief of depressive symptoms for at least two weeks in most patients [14,15]. Patients receiving DBS also experience relief of depressive symptoms in the majority of cases, and that lasts for at least one-year post-op. The role of VNS in TRD management is supported by small studies and metanalyses [16,17], all of which do show favorable outcomes in patients undergoing VNS. Lastly, psychotherapy alone has been shown to be no more effective that antidepressant therapy alone. However, psychotherapy in addition to traditional antidepressant therapy is far more efficacious than traditional antidepressant therapy alone in the treatment of TRD.

## Case presentation

The patient in this report is a 62-year-old female who has been diagnosed with generalized anxiety disorder and severe major depressive disorder; this patient admits to mainly experiencing major depressive episodes. She endorses trials of multiple antidepressant monotherapies, polytherapy, augmenting agents, psychotherapy, and transcranial magnetic stimulation, all of which have failed to produce substantial and/or sustained relief from her depressive symptoms. This patient’s history of failed antidepressant therapies meets the criteria for treatment-resistant depression.

This patient began experiencing depressive symptoms as a teenager, but she refused to seek help due to the stigma surrounding mental health at that time. She states the symptoms she experienced most frequently were irritability, low self-esteem, anger, rage, and frequent tearfulness. This patient admits to one suicide attempt as a teenager but states that her parents never sought treatment for her and that she was never hospitalized following that event. She has, however, been hospitalized twice as an adult, both instances occurring in the last two years. The first of these two hospitalizations was required for a rapid adjustment of medications, and the second was in response to the patient's reporting suicidal ideation with a plan. This patient admits to creating a plan for suicide on multiple different occasions throughout her life but has never been able to bring herself to carry these plans out. This patient’s past and current antidepressants as well as augmentation agents cover a wide range of pharmacological classes.

Antidepressants. SSRI’s: sertraline hydrochloride (current), desvenlafaxine (discontinued) and vortioxetine (discontinued); SNRI’s: venlafaxine (current); mirtazapine (current), bupropion (discontinued), and trazadone (discontinued); TCA’s: amitriptyline (current).

Augmentation agents. Atypical antipsychotics: aripiprazole (discontinued), olanzapine (discontinued); mood stabilizers: lithium (discontinued); benzodiazepines: clonazepam (current), mainly for anxiety; psychostimulants: dextroamphetamine and amphetamine combination salts (current).

This patient states that she has tried multiple antidepressant monotherapies but none of them were effective in treating her depressive symptoms. She states that lithium was one of the most effective augmentation therapies that she has been prescribed. Unfortunately, she had to discontinue it following the development of lithium-induced hypothyroidism. She is now being treated with levothyroxine. She was also unable to tolerate aripiprazole as she developed mild tardive dyskinesia (TD). Her TD is now being managed with valbenazine. Bupropion was also tried but was ineffective and was discontinued once dextroamphetamine and amphetamine combination salts were added to her treatment regimen.

This patient also underwent a full treatment course of TMS. After one session of TMS, the patient experienced a severe exacerbation of depressive symptoms; she was hospitalized for three days for suicidal ideation with a plan. TMS was put on hold until discharge. The entire course of treatment included 30 sessions conducted over a six-week period, with the patient undergoing five sessions a week at an outpatient TMS clinic. At the conclusion of the treatment course, the patient described a drastic relief of symptoms, claiming she felt better at that time than she had in many years. Unfortunately, about four months after her TMS treatment was completed, she had a relapse of depressive symptoms without suicidal ideation. As the patient began re-experiencing depressive symptoms, her dose of Effexor was increased; resuming lithium treatment, another round of TMS therapy and ECT were discussed at this time. However, considering the high cost of continuing TMS therapy and the short duration of symptom relief, the patient and her provider decided not to pursue another trial of TMS. This patient describes the feeling as though there was no hope left and that she was at the end of her rope with no more treatment options. It was at this time that dextroamphetamine and amphetamine combination salts of 10 mg were added to this patient’s treatment regimen. The prescribing physician was aware of psychostimulants being used in the management of TRD at this time but had never used them in practice.

Two weeks after dextroamphetamine and amphetamine combination salts were added to this patient's treatment regimen, she came into the office for follow-up. She came in exclaiming that she hadn’t felt as good as she does since she was a teenager. She stated that she began feeling the relief of symptoms within three days of starting dextroamphetamine and amphetamine combination salts and described her response as “a cloud being lifted off her mind.” She states that she can once again enjoy the activities that bring her the most happiness and fulfilment: spending time with her grandchildren, cooking, baking, and going to church. With tears in her eyes, this patient expressed an incredibly drastic relief of her depressive symptoms following the addition of dextroamphetamine and amphetamine combination salts to her treatment regimen. She states that she feels exponentially better with dextroamphetamine and amphetamine combination salts in her regimen than she ever did on antidepressants or after TMS.

At the six-month follow-up, the patient still endorsed a very significant relief of depressive symptoms. She stated that her mood is still very much improved, that she feels very stable on dextroamphetamine and amphetamine combination salts and even went as far as to call it “a wonder drug” for her. She has continued to maintain an increased activity level and is still able to partake in the activities that bring her the most joy in life. She denied any feelings of depression, thoughts of harm to herself or others, loss of appetite, nausea, vomiting, diarrhea, palpitations, tremors or seizures, problems sleeping, and increased anxiety. She endorsed a 15-pound weight loss since starting dextroamphetamine and amphetamine combination salts, an elevated mood, and increased motivation. She did state that she experienced feelings resembling mania when she first began taking dextroamphetamine and amphetamine combination salts, but that she had become much more stable and denied any further feelings of mania or manic episodes.

This patient was in full support of the writing of this case report as she hopes her success with dextroamphetamine and amphetamine combination salts augmentation will give other patients suffering from TRD hope for symptom remission.

## Discussion

Treatment-resistant depression is most commonly defined as the failure to achieve clinical remission of depressive symptoms following trials of two or more antidepressant medications from two different pharmacologic classes of adequate dosage, duration, and compliance [1]. TRD is estimated to effect 10-29% of patients diagnosed with major depressive disorder and bipolar depression; it appears to be a phenomenon most commonly effecting women aged 36 to 64 [2]. There are numerous treatment options for patients meeting the criteria for TRD, including various combinations of antidepressants, augmentation therapies, and non-pharmacological therapies such as electroconvulsive therapy, deep brain stimulation, transcranial magnetic stimulation, and vagus nerve stimulation. The current case report is of a 62-year-old female diagnosed with severe major depressive disorder and suggests that Adderall (dextroamphetamine and amphetamine) may be a safe and efficacious treatment option for patients meeting the criteria for treatment-resistant depression. In this patient, antidepressant monotherapy and polytherapy were ineffective. Various non-antidepressant augmentation therapies, as well as transcranial magnetic stimulation also failed to provide this patient with substantial and/or sustained remission of depressive symptoms.

One of the pharmacological options for augmentation is psychostimulants; this includes medications such as Adderall (dextroamphetamine-amphetamine), Ritalin (methylphenidate), and Vyvanse (lisdexamphetamine). Using psychostimulants in the management of TRD is not necessarily a novel idea; there is data and literature supporting the use of methylphenidate, modafinil, atomoxetine, and dextroamphetamine [3-12]. The data are somewhat mixed, showing cases where psychostimulant augmentation produced very little or no remission of symptoms [3,6,7] and some cases where psychostimulant augmentation was effective in achieving symptom remission [4-12]. The literature supporting the use of Adderall specifically is almost non-existent. We found only one uncontrolled trial and two case reports in which dextroamphetamine alone, not the combination of dextroamphetamine and amphetamine, was used in patients with TRD. The uncontrolled trial resulted in less than half of the patients receiving dextroamphetamine augmentation experiencing remission of depressive symptoms or a decrease in depressive symptoms [3]. The two case studies both positively support the use of dextroamphetamine in the management of TRD as both patients experienced remission of symptoms shortly after it was added to their antidepressant regimen [4,5].

Psychostimulants also present a relatively safe augmentation option with more benign adverse effects than other augmentation therapies. The side effect profiles of the current augmentation options are described above and should be strongly considered before adding augmentation therapy to a patient’s antidepressant regimen. Although most serious adverse effects are relatively rare, psychostimulants exhibit more mild side effects than other augmentation options like lithium, ketamine, antipsychotics, and dopaminergic agents such as amantadine and pramipexole. The patient presented in this case was forced to discontinue antipsychotic augmentation after she developed parkinsonism and tardive dyskinesia. Although she was willing to risk possibly worsening her TD symptoms and continue taking aripiprazole or olanzapine, psychostimulants offered an augmentation option with less detrimental adverse effects. This patient also had to discontinue lithium therapy following the development of clinical hypothyroidism. If psychostimulants are added to a patient’s medication regimen, the patient’s blood pressure must be monitored closely, a complete blood count (CBC) with differential should be obtained regularly, and the patient must be monitored for weight loss, seizures, exacerbation of tics, and comorbid stimulant addiction.

Once it is decided to use a psychostimulant as augmentation to a patient’s antidepressant regimen, it must then be decided which of the psychostimulants is going to be used. Although this decision is certainly for the patient and the provider to make together, the availability, cost, duration of action desired effects, and adverse effects of the individual psychostimulants must be considered. This then leads to a comparison of the psychostimulants mentioned above: Adderall (dextroamphetamine-amphetamine), Vyvanse (lisdexamphetamine), Ritalin (methylphenidate), atomoxetine, and modafinil. As our patient has experienced a drastic remission of depressive symptoms using Adderall, we will discuss the use of Adderall over the use of other psychostimulants.

Two of the most commonly used psychostimulants are Adderall (dexamphetamine-amphetamine) and Ritalin (methylphenidate). These medications share a mechanism of action, are available in short and long-acting preparations, and are also available as generic, and thus less costly, options. Although these two medications have similar durations of action, Ritalin (one to six hours sustained release (SR), 10-14 hours extended release (XR)) has a duration of action that is slightly more variable than that of Adderall (four to six hours SR, 10-12 hours XR). With regards to the extended-release options of both medications, Adderall offers a more consistent and reliable duration of action. There is one study that directly compares the effectiveness of Ritalin and Adderall in the treatment of attention-deficit/hyperactivity disorder (ADHD) [14]. This study found that Adderall was a much more potent option than Ritalin; Adderall was able to produce greater relief of ADHD symptoms at dose half as high as Ritalin. When considering the possible addictive nature of these drugs and the ability to offer effective relief of symptoms at lower doses, Adderall is a much safer option in this regard.

Vyvanse (lisdexamphetamine) is a prodrug of dextroamphetamine that is found in Adderall. Because it must first be metabolized to become active, the onset of action for Vyvanse is one to two hours after taking it. The two main ingredients of Adderall, dextroamphetamine and amphetamine, do not require metabolism to become active, therefore the onset of action for Adderall is typically 30 minutes. This shorter onset of action allows patients to experience relief of symptoms earlier in the day and much more quickly after taking Adderall than Vyvanse. Vyvanse also only comes in a preparation that lasts 13-14 hours after consumption; Adderall is available in long- and short-acting preparations. The long duration of action of these medications allows for consistent levels in the body throughout the day and a smoother onset and offset of effects. The short-acting preparation of Adderall, which lasts up to six hours, can also be used in patients not desiring such a long-action medication. A short-acting preparation also helps curb side effects such as insomnia, loss of appetite, etc. In addition to Adderall’s shorter onset of action and options for short or long-acting preparations, a generic option is also available that is significantly less costly than the brand name option; a generic option for Vyvanse will not be available until 2023 and, for the time being, is a more costly medication option than Adderall [15].

Atomoxetine and modafinil are non-amphetamine stimulant options for the augmentation of TRD therapy; these medications are most commonly used for the treatment of ADHD and narcolepsy. Compared to Adderall, Atomoxetine is much less likely to be abused and offers patients a non-amphetamine alternative for the treatment of their TRD. However, Atomoxetine is a significantly more expensive medication than Adderall [16] and it may take four to eight weeks to reach therapeutic levels, while Adderall is effective in relieving symptoms within 30 minutes to an hour and reaches therapeutic levels at a faster rate. Atomoxetine is also much more likely to lead to adverse effects such as insomnia, weight loss, and decreased appetite as its duration of action is up to 24 hours [17]. For these reasons, we believe Adderall is a superior option for psychostimulant augmentation than Atomoxetine.

Based on the literature we have reviewed and the current data available, modafinil is the psychostimulant augmentation option that is most comparable to Adderall for the treatment of depression. Both Adderall and modafinil have been shown to be effective in the management of TRD [4,5,11,13]. However, it appears as though modafinil offers a slightly safer option with a narrower side effect profile and a lower risk of abuse [18]. There are case reports and studies describing the possibility of Steven-Johnson syndrome in patients using modafinil. Although Adderall and modafinil are both available as generic options, Adderall is less costly than modafinil [18]. If managed properly and closely monitored, Adderall should be considered over modafinil because of the significantly lower cost of Adderall, especially in patients paying out of pocket.

After reviewing the literature comparing different psychostimulants, we believe that Adderall is currently the most efficacious and affordable option for psychostimulant augmentation in the management of TRD (Table 1).

**Table 1:** Pharmaceutical agents used, mechanisms of action, and adverse side effects. SIADH: syndrome of inappropriate antidiuretic hormone secretion, NMDA: N-methyl D-aspartate, GABA: gamma-aminobutyric acid.

Drug	Mechanism of action	Adverse effects
Selective serotonin reuptake inhibitors (SSRIs), i.e., fluoxetine, paroxetine, sertraline, citalopram	Inhibit reuptake of serotonin on the presynaptic neuron	Weight gain, drowsiness, sexual dysfunction, SIADH, and serotonin syndrome
Serotonin-norepinephrine reuptake inhibitors (SNRIs), i.e., venlafaxine, duloxetine	Inhibit the reuptake of serotonin and norepinephrine on the presynaptic neuron	Weight gain, drowsiness, sexual dysfunction, SIADH, and serotonin syndrome
Monoamine oxidase inhibitors (MAOIs), i.e., isocarboxazid, phenelzine, selegiline, moclobemide	Inhibit MAO-A (isocarboxazid, phenelzine, moclobemide) and MAO-B (selegiline) to prevent the metabolism of dopamine, norepinephrine, and serotonin	Dizziness, lightheadedness, changes in appetite, hypertensive crisis, serotonin syndrome
Tricyclic antidepressants (TCAs), i.e., amitriptyline, desipramine, imipramine	Inhibit presynaptic reuptake of serotonin and norepinephrine	Dry mouth, constipation, urinary retention, orthostatic hypotension, drowsiness, weight gain, arrhythmias, seizures
Trazadone	Agonizes postsynaptic serotonin receptors inhibit presynaptic serotonin reuptake and block alpha-2 receptors	Drowsiness, orthostatic hypotension, sexual dysfunction, priapism, serotonin syndrome
Mirtazapine	Antagonizes alpha-2 receptors and blocks 5-HT2 and 5-HT3 receptors to enhance 5-HT1 -mediated serotonergic transmission	Constipation, dry mouth, nausea, weight gain, drowsiness
Lithium	Stimulates NMDA receptors to increase glutamate activity in the brain	Weight gain, dry skin, hair loss, diabetes insipidus, tremor, ataxia, thyroid abnormalities, teratogenic
Buspirone	Stimulates postsynaptic serotonin receptors	Headaches, nausea, tinnitus, paresthesias, drowsiness
Benzodiazepines, i.e., diazepam, lorazepam	GABA receptor agonist	Drowsiness, confusion, dizziness, ataxia, CNS depression, dependence
Lamotrigine	Mood stabilizer; inactivates presynaptic sodium channels	Dizziness, drowsiness, vision changes, Steven-Johnson syndrome
Ketamine	NMDA receptor complex antagonist	Blurred vision, confusion, nausea, vomiting, hallucinations, dysphoria, respiratory depression
Dopaminergic agents, i.e., ropinirole, pramipexole	Stimulate D2 (ropinirole) and D3 (pramipexole) receptors in the caudate and putamen	Confusion, dizziness, drowsiness, hallucinations, orthostatic hypotension
Adderall (amphetamine/dextroamphetamine)	Inhibits reuptake and stimulates the release of dopamine and norepinephrine	Anorexia, weight loss, insomnia, dry mouth, GI upset, tremor, dependence
Vyvanse (lisdexamphetamine)	Inhibits reuptake and stimulates the release of dopamine and norepinephrine	Anorexia, weight loss, insomnia, dry mouth, GI upset, tremor, dependence
Ritalin (methylphenidate hydrochloride)	Inhibits reuptake and stimulates the release of dopamine and norepinephrine	Anorexia, weight loss, insomnia, dry mouth, GI upset, tremor, dependence
Atomoxetine	Inhibits norepinephrine reuptake in the frontal cortex	Anorexia, weight loss, insomnia, dry mouth, GI upset, tremor
Dextroamphetamine	Inhibits reuptake and stimulates the release of dopamine and norepinephrine	Anorexia, weight loss, insomnia, dry mouth, GI upset, tremor, dependence
Modafinil	Inhibits dopamine reuptake and stimulates orexin release (CNS stimulant)	Headache, nausea, nervousness, anorexia, paresthesias, Steven-Johnson syndrome

## Conclusions

Our case suggests that Adderall (dextroamphetamine and amphetamine) can be used as augmentation therapy in the management of patients meeting criteria for TRD and in cases where other non-pharmacological options have failed to produce remission of depressive symptoms. The patient in this case experienced a very drastic relief of depressive symptoms and an improvement in her quality of life within days of starting Adderall. After reviewing the literature and data available, Adderall has been shown to offer patients being treated for TRD an affordable, safe, and efficacious augmentation therapy option. Adderall also provides patients with the choice of long- or short-acting preparations as well as a medication that can begin relieving symptoms within two or three days of starting therapy. This patient was followed up with one month after starting her Adderall augmentation, and she states that her relief of symptoms has been sustained up until that point.

Further study into the use of Adderall in TRD is certainly warranted to test our clinical impressions. Prospective placebo-controlled trials comparing the use of Adderall and other stimulants like Vyvanse and Ritalin will be needed to further confirm the efficacy of Adderall in TRD augmentation therapy. We also suggest a trial directly comparing the efficacy of Modafinil and Adderall to further define the more efficacious option in the management of treatment-resistant depression.
